# Foveal Morphology Affects Self-Perceived Visual Function and Treatment Response in Neovascular Age-Related Macular Degeneration: A Cohort Study

**DOI:** 10.1371/journal.pone.0091227

**Published:** 2014-03-11

**Authors:** Yousif Subhi, Gitte Ø. Henningsen, Charlotte T. Larsen, Mette S. Sørensen, Torben L. Sørensen

**Affiliations:** 1 Clinical Eye Research Unit, Department of Ophthalmology, Copenhagen University Hospital Roskilde, Roskilde, Denmark; 2 University of Copenhagen, Copenhagen, Denmark; Zhongshan Ophthalmic Center, China

## Abstract

**Objectives:**

To investigate the relationship between foveal morphology and self-perceived visual function in patients with neovascular age-related macular degeneration (AMD) and whether foveal characteristics are associated with Ranibizumab treatment response on the self-perceived visual function.

**Methods:**

This prospective cohort study included patients with newly diagnosed neovascular AMD found eligible for treatment with Ranibizumab. Foveal morphology of both eyes was assessed using spectral-domain optical coherence tomography and all patients were interviewed using the 39-item National Eye Institute Visual Function Questionnaire (VFQ). Patients were re-interviewed 3 and 12 months after initiation of treatment with Ranibizumab. We evaluated foveal morphology at baseline in relation to VFQ scores at baseline and clinically meaningful changes in VFQ after 3 and 12 months.

**Results:**

VFQ scores correlated with central foveal thickness, central foveal thickness of neuroretina (CFN), foveal RPE elevation, foveal integrity of the photoreceptor inner segment/outer segment junction (IS/OS), and external limiting membrane. In a multiple linear regression model, only best-corrected visual acuity of the better eye (p<0.001) and the IS/OS status in the better eye (p = 0.012) remained significant (Adjusted R^2^ = 0.418). Lower baseline VFQ and a baseline CFN within 170–270 µm in the better eye were both associated with a clinically meaningful increase in the VFQ scores after 3 and 12 months. An absent foveal IS/OS band in the better eye was associated with a clinically meaningful decrease in the VFQ scores at 12 months.

**Conclusions:**

Foveal morphology in the better eye influences the self-perceived visual function in patients with neovascular AMD and possesses a predictive value for change in the self-perceived visual function at 3 and 12 months after initiation of treatment. These findings may help clinicians provide patients more individualized information of their disease and treatment prognosis from a patient-perceived point-of-view.

## Introduction

Age-related macular degeneration (AMD) is the leading cause of visual impairment among the elderly in the western world, and the late stages of the disease often lead to a severe negative impact on visual function. Hence, many patients experience a decreased ability to perform simple daily tasks and preserve social as well as mental function [1], [2]. Disease progression is traditionally monitored using visual acuity charts, which are unable to capture abstract dimensions of patient-perceived visual functioning [3]. Underscoring the importance of other measures of treatment effect than solely visual acuity, patient-perceived outcomes have become a common endpoint in clinical trials in medicine [4], and the National Eye Institute Visual Function Questionnaire (VFQ) is widely used and included in hallmark clinical studies in ophthalmology [5]–[8]. VFQ is validated in patients with AMD [9]–[11], and studies indicate that anti-vascular endothelial growth factor (anti-VEGF) treatment may maintain and in some cases improve the self-perceived visual function [5]–[8].

Emergence of high-resolution spectral-domain optical coherence tomography systems (OCT) have enabled detailed characterization of the foveal morphology in patients with neovascular AMD [12], [13]. Characteristics of foveal morphology assist in predicting treatment response of anti-VEGF in the management of wet AMD, e.g. smaller lesion size at baseline predicts of a good treatment response, and interruptions in certain hyperreflective bands such as the photoreceptor inner segment/outer segment junction (IS/OS) and external limiting membrane (ELM) predicts a bad treatment response [14]–[19]. Treatment response in these studies is measured as the change in visual acuity, which to the patient may be less relevant than the self-perceived visual function. Visual acuity and self-perceived visual function correlates only moderately and may not always go together [9], [20], [21]. Thus, we need studies investigating disease impact and prognosis from a patient-perceived point-of-view.

In this prospective cohort-study, we explored the relationship between baseline foveal characteristics and self-perceived visual function in patients with newly diagnosed neovascular AMD, and how baseline foveal characteristics are related to the self-perceived visual function after anti-VEGF treatment.

## Methods

### Study Population and Design

We included 200 consecutive patients newly diagnosed with neovascular AMD and eligible for treatment with intravitreal Ranibizumab from our outpatient clinic within the period of December 2009 and August 2011. All included patients were examined and interviewed at diagnosis and initiation of treatment, and re-examined and re-interviewed after 3 and 12 months. All patients received standard care. The Regional Committee of Ethics in Research of the Region of Zealand reviewed our study and waived further processing since the use of questionnaires within normal clinical practice does not, according to Danish law, require formal approval. Verbal and written informed consent was obtained from all participants prior to inclusion.

### Retinal Diagnosis and Ranibizumab Treatment

Best-corrected visual acuity (BCVA) was measured using Early Treatment Diabetic Retinopathy Study (ETDRS) chart. Retinal imaging was made using Spectral-Domain HRA-OCT (Heidelberg Engineering, Heidelberg, Germany) and images were examined using Heidelberg Eye Explorer version 1.7.0.0 (Heidelberg Engineering, Heidelberg, Germany). Fluorescein and indocyanine-green angiography were performed on all patients to confirm the diagnosis and to exclude other causes of maculopathy. In addition, we decided to exclude polypoidal chorioretinopathy due to its difference in clinical course and treatment response [22]. Initial treatment was given as a loading dose over three months with monthly intravitral injections of 0.5 mg Ranibizumab (Lucentis). Patients were re-examined at follow-ups 4 to 6 weeks after the third injection and the patients were prescribed additional injections based on signs of activity (retinal hemorrhages, presence of intra- and/or subretinal fluid) (PRN protocol) [23].

### Clinical Data

**Figure 1:**
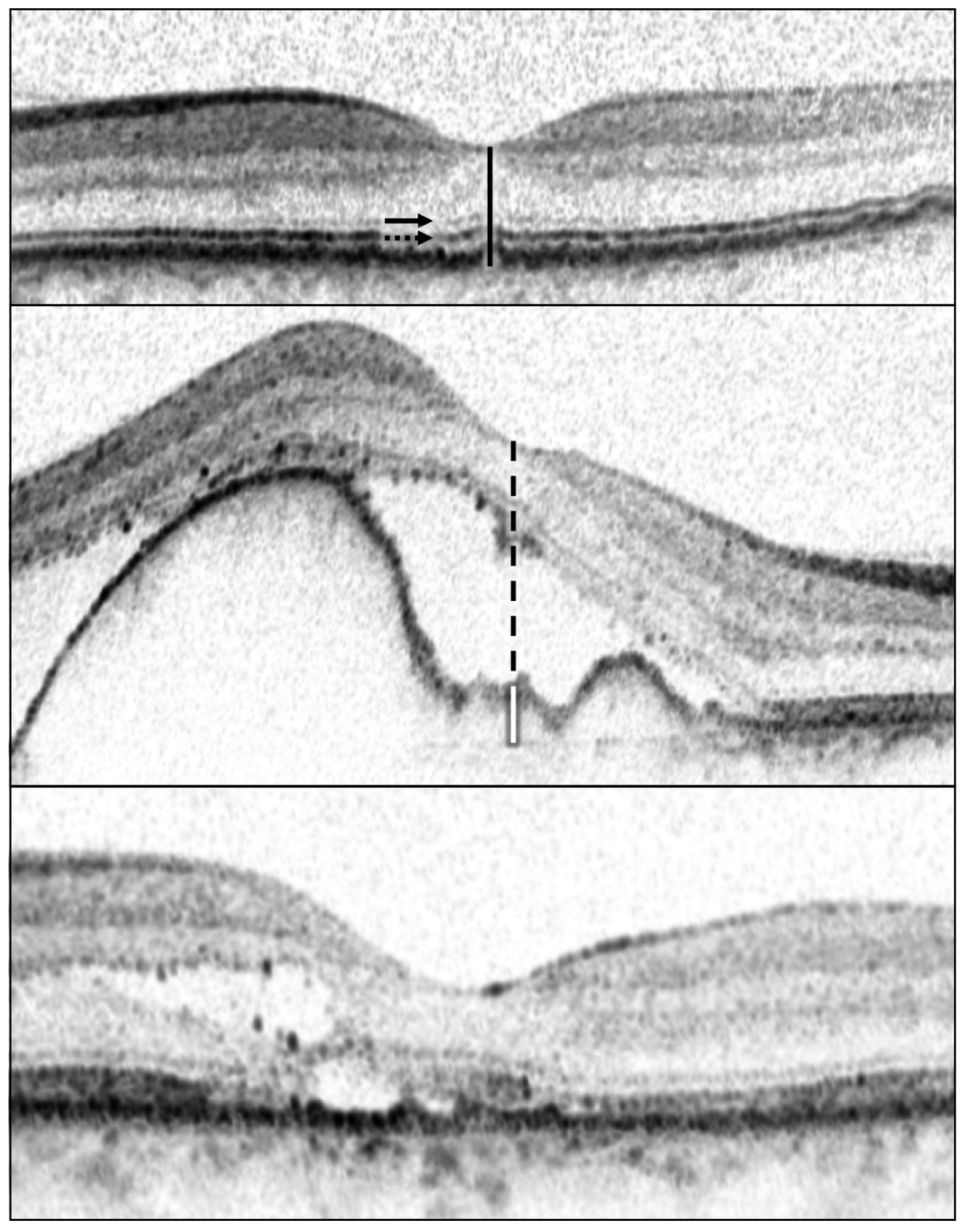
Optical coherence tomography images depicting measured foveal characteristics. We categorized the foveal status of external limiting membrane (ELM) (arrow) and inner segment/outer segment junction (IS/OS) (dotted arrow) as either complete, interrupted, or absent. We measured central foveal thickness (CFT) (black line), central foveal thickness of neuroretina including any serous detachment (CFN) (black dashed line), and central foveal thickness of RPE elevation in case of RPE elevation (CFE) (white line). (Top) Here, ELM and IS/OS were both complete, CFT and CFN were both 241 µm, and RPE was not elevated. (Middle) Here, ELM was complete, IS/OS was absent, CFT was 618 µm, CFN was 529 µm, RPE was elevated and CFE was 89 µm. (Bottom) Here, ELM and IS/OS were both interrupted, CFT and CFN were both 207 µm, and RPE was not elevated.

Horizontal cross-sectional Spectral-Domain HRA-OCT scans of the foveal region of both eyes were examined to: measure the central foveal thickness (CFT), measure the central foveal thickness of the neuroretina (including any serous detachment) (CFN), determine if RPE elevation is present and then measure the central foveal sub-RPE space (CFE), and categorize the foveal integrity of the IS/OS and the ELM as either complete, interrupted, or absent (**Figure 1**) [12].

### Assessment of Self-Perceived Visual Function

We utilized the Danish version of the 39-item VFQ, recently translated and validated for use in patients with AMD [9]. A trained interviewer presented the 39-item VFQ after the ophthalmic examination and diagnosis of neovascular AMD. The 39-item VFQ is composed by 39 questions grouped into 12 subscales, of which one is related to general health and the rest to different aspects of visual-functioning: general vision, ocular pain, near activities, distance activities, social function, mental health, role difficulties, dependency on others, driving, color vision, peripheral vision. Each subscale is scored between 0 (the worst) and 100 (the best), and the mean of all subscales (excluding general health) is the final composite VFQ score. As the Danish 39-item VFQ could not validate the subscales pertaining to ocular pain and driving, these subscales were not included in our study and therefore not part of the composite score. All interviews were performed without the presence of a relative or friend. The interviewer neither wore a uniform nor was part of the treating team, and followed a stringent predesigned interview guideline instructing adequate responses to patients when doubts arouse about a question. Furthermore, a questionnaire log was kept to record any deviations from normal protocol.

### Statistical Analysis

We correlated composite VFQ scores with BCVA of the better eye and BCVA of the worse eye using Spearman's Rank Correlation. Then, composite VFQ scores were transformed into normal distribution and used as the dependent variable in a backward multiple regression analysis. We used the Kolmogorov-Smirnov Test to check the transformed values for normal distribution. We used age, sex, BCVA of the better eye, and BCVA of the worse eye as co-variates in this model.

We analyzed the foveal morphological characteristics in the better eye, and when both eyes had same visual acuity, we repeated our analyses using the data from the right eye and the left eye separately. We found this to be an issue in three patients, and as these differences did not affect our results significantly, we simply choose to report the data from the right eye in these three cases. Examination of the horizontal cross-sectional scans were repeated by the examiner after three months to calculate intra-rater reliability using Cohen's unweighted kappa for categorical data and intraclass correlation coefficient (ICC) (two-way consistency model) for continuous data.

The relationship between continuous morphological data and VFQ scores were explored using scatter plots with a lowess curve. We correlated continuous morphological data with VFQ scores using Spearman's Rank Correlation. Patients were stratified into groups by RPE elevation, IS/OS status, and ELM status; and non-parametric tests (Kruskal-Wallis Test and Mann-Whitney-U Test) were used to compare VFQ scores.

We repeated our multiple regression analysis with the transformed composite VFQ scores and included foveal morphological characteristics in the better eye to investigate 1) whether inclusion of foveal characteristics would give a better model fit suggesting an additive value, and 2) whether we can identify and distinguish one or more foveal morphological characteristics which affects self-perceived visual function. Thus, we used the following co-variates: age, sex, BCVA of the better eye, BCVA of the worse eye, CFT in the better eye, CFN in the better eye, RPE elevation status in the better eye, IS/OS status in the better eye, and ELM status in the better eye. As the relationship between CFT and CFN values and VFQ scores was found to be non-linear in lowess curves, CFT and CFN values were converted into dummy variables with two possible values when used in the regression analysis: either 1) within the peak area defined as ≥170 and <270 µm or 2) outside of the peak area defined as <170 and ≥270 µm.

Changes in VFQ scores were grouped into one of three categories: *clinically meaningful increase*, *clinically meaningful decrease*, and *no clinically meaningful change*. A 5-point or more change in the composite VFQ score is regarded as a clinically meaningful change [20], [21]. We compared these changes in relation to baseline values including foveal morphological characteristics using Kruskal-Wallis Test and Chi-square Test (Fischer's Exact Test when dealing with small numbers). We then used a multinomial logistic regression to investigate whether any baseline characteristics were associated with a *clinically meaningful increase* or a *clinically meaningful decrease* in composite VFQ score at 3 and 12 months, using the *no clinically meaningful change* category as the reference category. We included the following baseline characteristics: composite VFQ score, CFT in the better eye, CFN in the better eye, RPE elevation status in the better eye, IS/OS status in the better eye, ELM status in the better eye, BCVA of the better eye, and BCVA of the worse eye. Due to the inverse U-shaped relation of CFT and CFN in the better eye with VFQ scores, and small numbers in the <150 µm groups, CFT and CFN in the better eye were included as dummy variables with two possible values: either 1) within the peak area defined as ≥170 and <270 µm or 2) outside of the peak area defined as <170 and ≥270 µm.

A p-value below 0.05 is considered significant, which was Bonferroni-adjusted to a p-value below 0.005 when testing VFQ subscores. Data were analyzed using SPSS 20 (IBM, Chicago, IL, USA) and Prism 6 (GraphPad Software, La Jolla, CA, USA).

## Results

**Figure 2:**
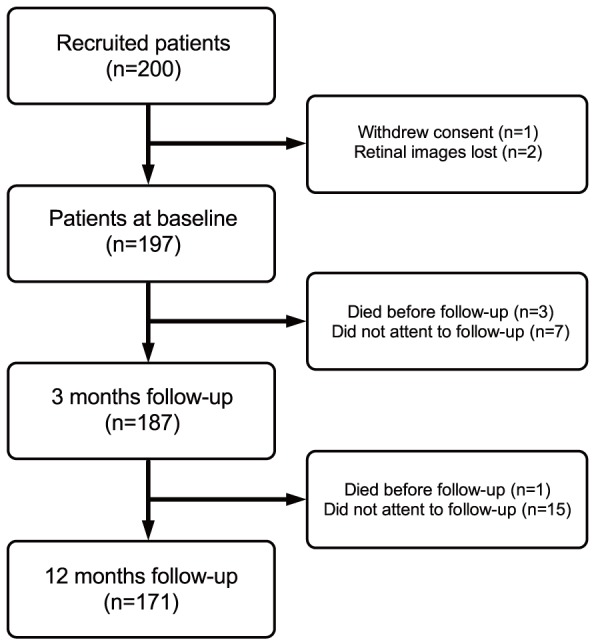
Study flow diagram with number of participants at each stage and reasons for non-participation.

We enrolled 200 patients in our cohort, but one withdrew consent and in two patients the OCTs from one time-point were lost, leaving 197 patients. Patient characteristics are summarized in **Table 1**. Of the included patients, 187 completed the 3 months follow-up, and 171 completed the 12 months follow-up (**Figure 2**).

**Table 1:** Patient characteristics.

Characteristics (n = 197)	
Age, median (IQR), years	79 (74–84)
Sex (male), n (%)	61 (31)
Neovascular AMD in eyes, n (%)	
In better eye only	65 (33)
In worse eye only	115 (58)
In both eyes	17 (9)

Composite VFQ scores correlated stronger with BCVA of the better eye (ρ = 0.605; p<.001) compared to BCVA of the worse eye (ρ = .405; p<0.001). In our multiple regression analysis, we found BCVA of the better eye to be a significant co-variate (p<0.001), but not BCVA of the worse eye (p = 0.362), age (p = 0.526), and sex (p = 0.850) (Adjusted R^2^ = 0.398).

### Self-Perceived Visual Function and Foveal Morphology in the Better Eye

Foveal morphological characteristics in the better eye are summarized in **Table 2**. Intra-rater reliability was high for all measured aspects of foveal characteristics: CFT in the better eye (ICC = 0.996), CFN in the better eye (ICC = 0.983), RPE elevation in the better eye (κ = 1.000), IS/OS status in the better eye (κ = 0.964), ELM status in the better eye (κ = 0.935).

**Table 2:** Foveal morphological characteristics in the better eye of study participants.

Characteristics (n = 197)	
CFT, median (IQR), µm	261 (223–375)
CFN, median (IQR), µm	237 (202–281)
RPE elevation, n (%)	
Not present	121 (61)
Present	76 (39)
CFE, median (IQR), µm	102 (56–173)
IS/OS status, n (%)	
Complete	84 (43)
Interrupted	40 (20)
Absent	73 (37)
ELM status, n (%)	
Complete	95 (48)
Interrupted	40 (20)
Absent	62 (32)

Abbreviations used: CFT  =  total central foveal thickness, IQR  =  interquartile range, CFN  =  central foveal thickness of the neuroretina, RPE  =  retinal pigment epithelium, CFE  =  central foveal thickness of the retinal pigment epithelium elevation, IS/OS  =  inner segment/outer segment junction, ELM  =  external limiting membrane.

**Figure 3:**
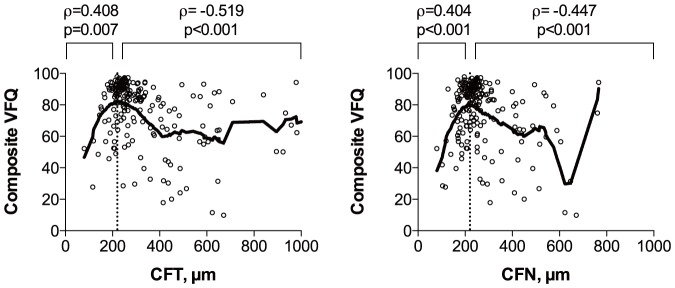
Composite VFQ scores in relation to foveal thickness in the better eye. Scatterplots with a lowess curve (thick line) to explore the relationship between composite VFQ scores and total central foveal thickness (CFT) in the better eye (left) and central foveal thickness of the neuroretina (CFN) in the better eye (right). Both CFT and CFN correlated with composite VFQ scores in an inverted U-shaped fashion peaking at approximately 220 µm (dotted line). ρ =  Spearman's rank correlation coefficient, p =  p-value.

**Figure 4:**
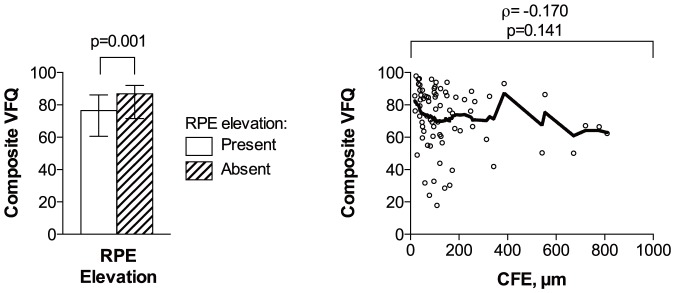
Composite VFQ scores in relation to retinal pigment epithelium status. Composite VFQ scores are compared between patients with or without retinal pigment epithelium (RPE) elevation in better eye (left). A scatterplot with a lowess curve (thick line) is used to explore the relationship between composite VFQ scores and central foveal thickness of the RPE elevation (CFE) (n = 76) (right). RPE elevation in the better eye was associated with lower composite VFQ scores; however, the level of RPE elevation — measured as CFE — did not correlate significantly with composite VFQ scores. The height of bars represents the median, and the whiskers represent the interquartile range. ρ =  Spearman's rank correlation coefficient; p =  p-value.

**Figure 5:**
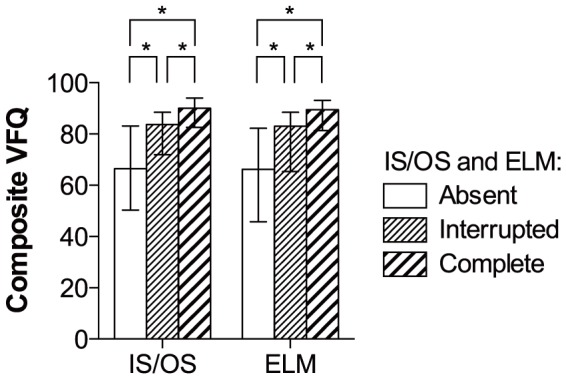
Comparison of composite VFQ scores between patients stratified by the foveal status of IS/OS and ELM in the better eye. A better IS/OS or ELM status in the better eye was associated with a higher composite VFQ score. *  =  significant (p<0.05).

CFT and CFN in the better eye correlated with composite VFQ scores in an inverted U-shaped fashion peaking at approximately 220 µm (**Figure 3**). RPE elevation in the better eye was associated with lower composite VFQ scores, but CFE in the better eye did not correlate with composite VFQ scores (**Figure 4**). Better IS/OS and ELM status in the better eye were both associated with higher composite VFQ scores (**Figure 5**).

**Figure 6:**
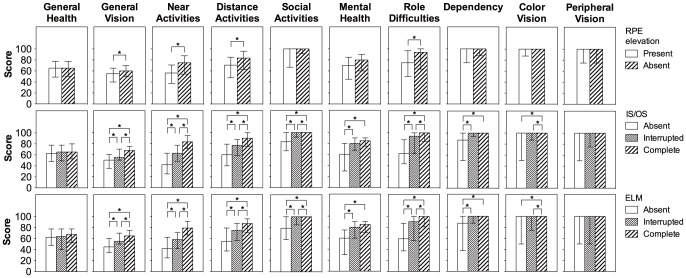
VFQ subscores in relation to foveal status of IS/OS, ELM, and presence of RPE elevation. Presence of retinal pigment epithelium (RPE) elevation was associated with lower general vision, lower near activities, lower distance activities, and lower role difficulties (top). Better inner segment/outer segment junction (IS/OS) (middle) and better external limiting membrane (ELM) (bottom) status were both associated with higher general vision, near activities, distance activities, social activities, mental health, role difficulties, dependency, and color vision. *  =  significant (p<0.005).

Low CFT values (<220 µm) in the better eye correlated positively with near activities, mental health, and dependency; and high CFT values (≥220 µm) correlated negatively with general vision, near activities, distance activities, social function, mental health, role difficulties, dependency, and color vision (**Table 3**). Low CFN values (<220 µm) in the better eye correlated positively with general vision, near activities, distance activities, social function, mental health, role difficulties, and dependency; and high CFN values (≥220 µm) correlated negatively with general vision, near activities, distance activities, social function, mental health, role difficulties, dependency, and color vision (**Table 3**). Presence of RPE elevation in the better eye was associated with lower near vision (**Figure 6**), but CFE did not correlate with any subscores (**Table 3**). Better IS/OS and ELM status in the better eye were both associated with better general vision, near activities, distance activities, social function, mental health, role difficulties, dependency, and color vision (**Figure 6**).

**Table 3:** Spearman's rank correlation coefficient of the correlation between foveal thickness parameters in the better eye and Visual Function Questionnaire subscores.

	CFT <220 µm (n = 43)	CFT ≥220 µm (n = 154)	CFN <220 µm (n = 74)	CFN ≥220 µm (n = 123)	CFE (n = 76)
General health	0.110	−0.103	−0.013	−0.054	−0.064
General vision	0.350	−0.524^a^	0.268	−0.457^a^	−0.094
Near activities	0.382	−0.560^a^	0.449^a^	−0.498^a^	−0.213
Distance activities	0.357	−0.489^a^	0.325^b^	−0.374^a^	−0.221
Social function	0.282	−0.457^a^	0.265	−0.488^a^	−0.067
Mental health	0.425^b^	−0.349^a^	0.398^a^	−0.337^a^	−0.060
Role difficulties	0.365	−0.450^a^	0.397^a^	−0.381^a^	−0.185
Dependency	0.285	−0.379^a^	0.374^c^	−0.343^a^	−0.157
Color vision	0.293	−0.312^a^	0.297	−0.356^a^	−0.123
Peripheral vision	0.355	−0.150	0.261	−0.196	0.057

Abbreviations used: CFT  =  total central foveal thickness, CFN  =  central foveal thickness of the neuroretina, CFE  =  central foveal thickness of the retinal pigment epithelium elevation.

a Significant correlation, p<0.001.

b Significant correlation, p = 0.005.

c Significant correlation, p = 0.001.

Including foveal characteristics in our multiple regression analysis gave a better model fit (Adjusted R^2^ = 0.418) compared to the previous model which only included age, sex, BCVA of the better eye, and BCVA of the worse eye. In this model, BCVA of the better eye (p<0.001) and IS/OS status in the better eye (p = 0.005) were significant, while the other co-variates did not reach significance: age (p = 0.592), sex (p = 0.685), BCVA of the worse eye (p = 0.501), CFT in the better eye (p = 0.702), CFN in the better eye (p = 0.343), RPE elevation status in the better eye (p = 0.887), and ELM status in the better eye (p = 0.633).

### Self-Perceived Visual Function and Morphology Status in the Worse Eye

**Figure 7:**
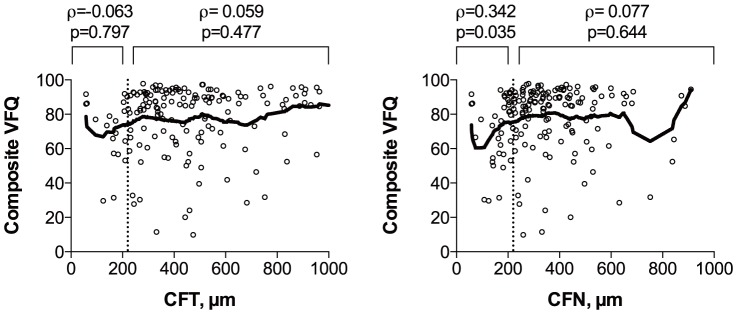
Composite VFQ scores in relation to foveal thickness in patients with neovascular AMD in their worse eye. Scatterplots with a lowess curve (thick line) to explore the relationship between composite VFQ scores and total central foveal thickness (CFT) in the worse eye (left) and central foveal thickness of the neuroretina (CFN) in the worse eye (right). We divided the cases into groups at 220 µm (dotted line) as previously and found no correlation between VFQ and CFT or CFN ≥220 µm, and we found a small correlation between VFQ and CFN <220 µm. ρ =  Spearman's rank correlation coefficient, p =  p-value.

**Figure 8:**
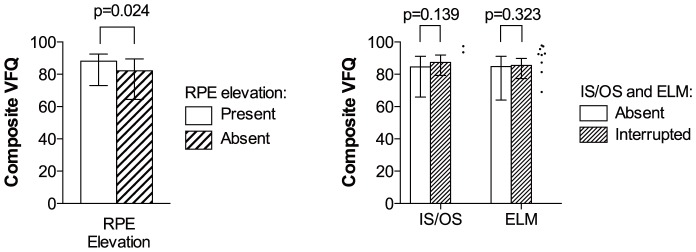
Comparison of composite VFQ scores in patients with neovascular AMD in their worse eye, stratified by the foveal status of RPE elevation, IS/OS and ELM. Composite VFQ scores were lower in patients with elevated retinal pigment epithelium (RPE) (left). IS/OS or ELM status did not affect composite VFQ scores significantly (right). Due to very few cases, worse eyes with complete IS/OS (n = 2) and complete ELM status (n = 9) are shown as dots and not included in statistical analyses. p =  p-value.

We re-ran our analyses on patients only unilaterally affected by neovascular AMD in order to evaluate the impact of worse eye morphology status on self-perceived visual function. We did not find a correlation between composite VFQ scores and CFT or CFN (Figure 7). Elevated RPE in the worse eye had a significant impact on composite VFQ scores (Figure 8), but the extent of RPE elevation did not correlate with composite VFQ scores (ρ = −0.153, p = 0.159, Spearman's Rank Correlation). Status of IS/OS or ELM in the worse eye did not contribute to composite VFQ scores (Figure 8).

### Clinically Meaningful Changes in Self-Perceived Visual Function at 3 and 12 Months

BCVA of the treated eyes did not change significantly during the study period; however, composite VFQ scores increased from baseline to 3 months, but fell at 12 months to a level similar to baseline (**Table 4**). Clinically meaningful changes in composite VFQ at 3 months were associated with baseline composite VFQ score, baseline BCVA of the better eye, baseline IS/OS status in the better eye, and baseline ELM status in the better eye (**Table 5**). Clinically meaningful changes in composite VFQ at 12 months were associated with baseline composite VFQ scores, baseline BCVA of the better eye, baseline BCVA of the worse eye, baseline CFT in the better eye, and baseline IS/OS status in the better eye (**Table 5**). When these values were included in multinomial logistic regression analyses, we found a clinically meaningful increase at 3 months to be associated with a lower baseline composite VFQ (p<0.001) and a baseline CFN <170 µm or ≥270 µm in the better eye (p = 0.043); a clinically meaningful increase at 12 months to be associated with a lower baseline composite VFQ (p<0.001); a clinically meaningful decrease at 3 months not to be associated with any baseline variables; and a clinically meaningful decrease to be associated with an absent IS/OS line in the better eye at baseline (p = 0.018) (**Table 6**).

**Table 4:** Best-corrected visual acuity of the treated eye and composite Visual Function Questionnaire scores throughout the study period.

	Baseline (197 patients, 214 eyes)	3 Months Follow-up (187 patients, 202 eyes)	12 Months Follow-up (n = 171 patients, 186 eyes)
BCVA of the treated eye, median (IQR), ETDRS letters	59 (48–70)	62 (48–72)	62 (47–72)
Composite VFQ Scores, median (IQR)^a^	84 (67–91)	85 (68–92)^b^	83 (65–90)^c^

Abbreviations used: BCVA  =  best-corrected visual acuity, IQR  =  interquartile range, ETDRS  =  Early Treatment Diabetic Retinopathy Study, VFQ  =  Visual Function Questionnaire.

a Significant change throughout the study period, p<0.001 in Friedman's two-way analysis of variance by ranks.

b Significant change between baseline and 3 months follow-up, p = 0.004 in Wilcoxon signed rank test.

c Significant change between 3 and 12 months follow-up, p<0.001 in Wilcoxon signed rank test.

**Table 5:** Baseline characteristics in relation to clinical meaningful changes in composite Visual Function Questionnaire scores at 3 and 12 months.

	3 months follow-up				12 months follow-up			
	Increase (n = 62)	No change (n = 86)	Decrease (n = 39)	P-value	Increase (n = 41)	No change (n = 81)	Decrease (n = 49)	P-value
Age, median (IQR), years	77 (73–82)	79 (75–85)	80 (76–84)	0.22^a^	78 (74–82)	78 (74–83)	79 (75–84)	0.43^a^
Sex (male), n (%)	20 (32)	24 (28)	14 (36)	0.65^b^	13 (32)	22 (27)	15 (31)	0.85^b^
Composite VFQ score	72 (57–82)	89 (81–93)	83 (60–91)	<0.001^a^	69 (53–80)	89 (79–93)	86 (69–90)	<0.001^a^
BCVA of the better eye, ETDRS letters	74 (56–81)	78 (70–85)	71 (63–78)	0.03^a^	76 (54–80)	78 (71–85)	71 (64–78)	0.005^a^
BCVA of the worse eye, ETDRS letters	48 (22–66)	50 (31–67)	48 (29–57)	0.48^a^	50 (22–67)	55 (41–68)	46 (26–60)	0.05^a^
**Foveal morphology in the better eye**								
CFT, n (%)								
<170 µm	7 (11)	2 (2)	1 (3)		6 (15)	1 (1)	2 (4)	
≥170 µm and <270 µm	27 (44)	47 (55)	16 (41)	0.10^c^	20 (49)	48 (59)	18 (37)	0.004^c^
≥270 µm	28 (45)	37 (43)	22 (56)		15 (37)	32 (40)	29 (59)	
CFN, n (%)								
<170 µm	8 (13)	4 (5)	4 (10)		7 (17)	3 (4)	4 (8)	
≥170 µm and <270 µm	38 (61)	59 (69)	21 (54)	0.26^c^	24 (59)	59 (73)	29 (59)	0.09^c^
≥270 µm	16 (26)	23 (27)	14 (36)		10 (24)	19 (23)	16 (33)	
RPE elevation, n (%)								
Not present	39 (63)	54 (63)	21 (54)	0.59^b^	26 (63)	50 (62)	27 (55)	0.68^b^
Present	23 (37)	32 (37)	18 (46)		15 (37)	31 (38)	22 (45)	
IS/OS status, n (%)								
Complete	20 (32)	48 (56)	9 (23)		16 (39)	43 (53)	13 (27)	
Interrupted	16 (26)	15 (17)	8 (21)	0.002^b^	8 (20)	17 (21)	12 (24)	0.03^b^
Absent	26 (42)	23 (27)	22 (56)		17 (41)	21 (26)	24 (49)	
ELM status, n (%)								
Complete	25 (40)	51 (59)	12 (31)		18 (44)	46 (57)	19 (39)	
Interrupted	15 (24)	20 (23)	9 (23)	0.03^b^	9 (22)	16 (20)	11 (22)	0.29^b^
Absent	22 (35)	38 (44)	18 (46)		14 (34)	19 (23)	19 (39)	

Abbreviations used: IQR  =  interquartile range, VFQ  =  Visual Function Questionnaire, BCVA  =  best-corrected visual acuity, ETDRS  =  Early Treatment Diabetic Retinopathy Study, CFT  =  total central foveal thickness, CFN  =  central foveal thickness of the neuroretina, RPE  =  retinal pigment epithelium, IS/OS  =  inner segment/outer segment junction, ELM  =  external limiting membrane.

a Kruskal-Wallis test.

b Chi-square test.

c Fischer's Exact Test.

**Table 6:** Multinomial logistic regression on clinical meaningful changes in composite Visual Function Questionnaire scores at 3 and 12 months using baseline characteristics.

	3 months follow-up				12 months follow-up			
	Increase (n = 62)		Decrease (n = 39)		Increase (n = 41)		Decrease (n = 49)	
	OR (95% CI)	P-value	OR (95% CI)	P-value	OR (95% CI)	P-value	OR (95% CI)	P-value
Age	.94 (.80–1.0)	0.06	.97 (.91–1.0)	0.37	.96 (.89–1.0)	0.21	.98 (.92–1.0)	0.62
Sex (male)	.12 (.50–3.0)	0.67	1.8 (.69–4.6)	0.24	1.4 (.50–3.7)	0.55	.83 (.33–2.1)	0.69
Composite VFQ score	.92 (.89–.95)	<0.001	.99 (.95–1.0)	0.42	.93 (.90–.96)	<0.001	1.0 (.98–1.1)	0.31
BCVA of the better eye	1.0 (.99–1.1)	0.09	1.0 (.97–1.1)	0.49	.99 (.94–1.0)	0.66	.99 (.94–1.0)	0.77
BCVA of the worse eye	1.0 (.99–1.0)	0.29	1.0 (.98–1.0)	0.77	1.0 (.99–1.0)	0.26	.98 (.96–1.0)	0.09
**Foveal morphology in the better eye**								
CFT								
≥170 µm and <270 µm	1.0	-	1.0	-	1.0	-	1.0	-
<170 µm or ≥270 µm	2.5 (.66–9.3)	0.18	.57 (.15–2.2)	0.41	.93 (.21–4.2)	0.92	2.4 (.67–8.5)	0.18
CFN								
≥170 µm and <270 µm	1.0	-	1.0	-	1.0	-	1.0	-
<170 µm or ≥270 µm	.27 (.08–.96)	0.04	1.5 (4.7–5.0)	0.48	.89 (.21–3.8)	0.88	.81 (.27–2.5)	0.71
RPE elevation								
Not present	1.0	-	1.0	-	1.0	-	1.0	-
Present	.36 (.13–1.0)	0.06	.86 (.31–2.4)	0.77	.56 (.19–1.7)	0.31	.55 (.20–1.5)	0.23
IS/OS status								
Complete	1.0	-	1.0	-	1.0	-	1.0	-
Interrupted	4.8 (.76–31)	0.09	2.1 (.27–16)	0.48	.69 (.08–5.9)	0.74	5.1 (.90–29)	0.07
Absent	2.6 (.29–23)	0.40	6.2 (.76–50)	0.09	1.0 (.08–14)	0.994	14 (1.6–118)	0.02
ELM status								
Complete	1.0	-	1.0	-	1.0	-	1.0	-
Interrupted	.94 (.15–5.9)	0.95	1.9 (.27–14)	0.51	1.6 (.19–13)	0.67	.39 (.07–2.1)	0.28
Absent	1.2 (.13–11)	0.88	1.6 (.20–13)	0.66	.67 (.05–9.8)	0.77	.17 (.02–1.5)	0.11

Values are in odds ratio and 95% confidence interval. Age (in years), composite VFQ score, BCVA of the better eye (in ETDRS letters), and BCVA of the worse eye (in ETDRS letters) are included as continuous co-variates. The no clinical meaningful change group was used as the reference category. Intercepts are not shown.

Abbreviations used: OR  =  odds ratio, CI  =  confidence interval, VFQ  =  Visual Function Questionnaire, BCVA  =  best-corrected visual acuity, CFT  =  total central foveal thickness, CFN  =  central foveal thickness of the neuroretina, RPE  =  retinal pigment epithelium, IS/OS  =  inner segment/outer segment junction, ELM  =  external limiting membrane.

## Discussion

BCVA of the better eye correlated with the VFQ scores at a level similar to previous studies (ρ = 0.605), which indicates an only moderate level of correlation between BCVA and self-perceived visual function [9], [20], [24]. Hence, the self-perceived visual function is not just another measure of BCVA, but an important addition to the overall assessment of treatment response. Previous studies have mainly focused on the predictive value of retinal morphology on visual acuity, and some have investigated self-perceived visual function in relation to treatment response. In our prospective study, we investigated the relationship between foveal morphology and self-perceived visual function, and the predictive value of foveal morphology on changes in self-perceived visual function. We found IS/OS status of the better eye to contribute to the self-perceived visual function independent of BCVA of the better eye, and we identified three baseline factors which were associated with clinically meaningful changes in self-perceived visual function.

Foveal morphology and its effect on self-perceived visual function has not previously been studied; however, some studies have reported on foveal morphology and its impact on visual acuity. In eyes with neovascular AMD, foveal thickness correlated in a V-shaped fashion with visual acuity measured in logMAR, and IS/OS and ELM status had a significant impact on visual acuity in patients with neovascular AMD [12], [13]. The extent of foveal IS/OS distruption has also been linked to lower visual acuity in eyes with dry age-related macular degeneration and neovascular AMD [14], [25]. Similar findings are observed in eyes with other retinal diseases, so there is strong data supporting that photoreceptor integrity, represented by the IS/OS layer, has a significant impact on the visual acuity [26]–[28]. In our study, we found foveal thickness and foveal integrity of IS/OS and ELM to be related to the self-perceived visual function. However, visual acuity and self-perceived visual function correlates only moderately and does not always go together, and therefore one cannot assume that the impact of foveal morphology on the self-perceived visual function is equal to findings on visual acuity. One particularly interesting finding in our study is that the IS/OS status in the better eye contributed independently of the BCVA of the better eye to the self-perceived visual function, which indicates that visual acuity does not always capture the self-perceived visual function. This is perhaps unsurprising, as the self-perceived visual function measured by the VFQ reflects the patient-experienced visual function and vision-related quality-of-life, and contributes with a more complex and subjective dimension of visual function compared to visual acuity [29]. This is exemplified by one study which showed that anti-VEGF treatment of eyes with neovascular AMD could improve the ability to read although treatment did not improve the visual acuity [30]. In another study, reading was significantly impaired in patients with subretinal fibrosis after choroidal neovascularization, and underestimated when only considering the visual acuity [31].

Ranibizumab phase III trials MARINA and ANCHOR showed a significant increase in visual acuity after 3, 12, and 24 months [5]. These studies also investigated the self-perceived visual function using the VFQ-25 and found significant higher VFQ scores at 3, 12, and 24 months, excluding cases in the ANCHOR study, where the study eye was the worse eye [5]. Unlike these controlled clinical trials, studies based on daily clinical practice tend to show a lower effect of treatment, although still much better compared to the sham group in MARINA and ANCHOR studies [6], [23], [32], [33]. Less is known on how the self-perceived visual function changes in daily clinical practice. Using the VFQ-39, we found that the VFQ scores increased significantly at 3 months, which returned to a level similar to baseline after 12 months. This is in line with the only other study, which also found a minor increase in VFQ-25 composite score after 3 months, which returned to a level similar to baseline after one year [6]. In the majority of our patients, changes in VFQ scores were small and studies suggest that a clinically meaningful change in the VFQ score is at least 4 to 6 points [20], [21]. No previous studies have investigated predictive factors of clinical meaningful change in VFQ; however, certain baseline predictors have been associated with a clinically meaningful improvement in visual acuity (≥15 letters) 1 year after anti-VEGF treatment: lower baseline BCVA and smaller lesion size at baseline is associated with an improvement in visual acuity at 1 year [15]–[17]. Also, IS/OS and ELM status at baseline are shown to influence visual acuity after 1 year of treatment [14], [18], [19]. When we used a 5-point change limit to identify cases with a clinically meaningful change at 3 and 12 months, we could observe several associated baseline characteristics: baseline VFQ score, baseline BCVA, baseline CFT value, baseline IS/OS status, and baseline ELM status. In our multinomial logistic regression analysis, we found a lower baseline VFQ to be positively associated with an increase at 3 and 12 months, a baseline CFN <170 µm or ≥270 µm in the better eye to be negatively associated with an increase at 12 months, and an absent IS/OS line in the better eye to be positively associated with a decrease at 12 months. Visual acuity and self-perceived visual function does not necessarily go together; however, in the prediction of clinically meaningful changes in self-perceived visual function, we find similar factors to possess a predictive value.

This study has several strengths. We used the VFQ-39 with trained interviewers, which provide more valid and accurate data in patients with neovascular AMD [9]–[11]. Validity of the VFQ has been questioned as some VFQ subscales are found to be psychometrically flawed [34]. Studies on the validity of the VFQ and its subscales have determined that the VFQ-25, including the 14 question appendix (VFQ-39) and excluding subscales concerning ocular pain and driving, is valid when studying Danish patients with AMD [9]–[11], [35]. We included patients with neovascular AMD consecutively to minimize any selection bias. Finally, our clinic receives all patients in Region Zealand (∼820.000 citizens) for neovascular AMD diagnosis and treatment without any self-payment; therefore, our results are based on a general non-selected population, which strengthens its relevance for daily clinical practice. One should also note the limitations of our study. BCVA of the worse eye did not contribute significantly to the self-perceived visual function. This is not surprising, especially in light of a previous study by Rubin et al., which investigated the visual acuity in the elderly and found that only the better eye is of importance to vision-related tasks, such as reading and face-discrimination, suggesting that the contribution from the worse eye may be small at most [36]. Our similar findings confirm that the better eye is the primary contributor to the self-perceived visual function. However, we found that elevated RPE in the worse eye contributed to the composite VFQ scores, which suggest that foveal morphology in the worse eye may influence the self-perceived visual function albeit subtler and less significant compared to the better eye. The details on how and when the foveal morphology of the worse eye influences the self-perceived visual function remain to be investigated. The VFQ scores range from 0 to 100, which limit any prediction of clinically meaningful changes near the range borders due to flooring or ceiling effect. Another important aspect is our sample size, which may large and sufficient to reveal factors with a high contribution to the self-perceived visual function, but may be too small for detecting factors with a more subtle contribution to the self-perceived visual function. For example, clinically meaningful decrease at 12 months is almost significantly associated with an interrupted IS/OS line (OR 5.1, p = 0.07); therefore, future studies should consider larger sample sizes when investigating clinically meaningful changes, as anti-VEGF treatment may be able to preserve the self-perceived visual function in the majority of patients with neovascular AMD.

In conclusion, foveal morphology is related to the self-perceived visual function, and the foveal IS/OS status in the better eye may influence on the self-perceived visual function independently of visual acuity. Lower VFQ score and baseline CFN in the better eye within 170 µm to 270 µm are both associated with a clinically meaningful increase in the self-perceived visual function. Absent foveal IS/OS line in the better eye is associated with a clinically meaningful decrease in the self-perceived visual function 12 months after treatment start. These associations should not discourage the use of anti-VEGF in patients with an absent IS/OS line, as some patients may experience a clinically meaningful increase. Instead, these novel findings can help clinicians provide patients more individualized information on the prognosis and what to expect on a vision-related quality-of-life perspective. Prediction of patients at risk of impaired vision-related quality-of-life may help us better target support and rehabilitation, especially considering the increasing number of elderly and the economical impact of the future prevalence of neovascular AMD [37]–[39]. Future studies need to investigate which aspects of the self-perceived visual function are affected, how treatment response on foveal morphology affects the self-perceived visual function, and whether identification of patients at risk may help us more rationally target eye-related care and rehabilitation.
